# The Clinical Usefulness of Tuberculin Skin Test versus Interferon-Gamma Release Assays for Diagnosis of Latent Tuberculosis in HIV Patients: A Meta-Analysis

**DOI:** 10.1371/journal.pone.0161983

**Published:** 2016-09-13

**Authors:** Erfan Ayubi, Amin Doosti-Irani, Ali Sanjari Moghaddam, Mohadeseh Sani, Milad Nazarzadeh, Ehsan Mostafavi

**Affiliations:** 1 Department of Epidemiology, School of Public Health, Shahid Beheshti University of Medical Sciences, Tehran, Iran; 2 Department of Epidemiology, Pasteur Institute of Iran, Tehran, Iran; 3 Department of Epidemiology, School of Public Health, Tehran University of Medical sciences, Tehran, Iran; 4 School of Medicine, Shahid Beheshti University of Medical Sciences, Tehran, Iran; 5 School of Medicine, Zabol University of Medical Sciences, Zabol, Iran; 6 The collaboration center of meta-analysis research (ccMETA), Iranian Research Center on Healthy Aging, Sabzevar University of Medical Sciences, Sabzevar, Iran; 7 Research Center for Emerging and Reemerging infectious diseases (Akanlu), Hamadan, Iran; Chinese Academy of Medical Sciences and Peking Union Medical College, CHINA

## Abstract

**Background:**

Accurate diagnosis of latent tuberculosis infection (LTBI) is becoming increasingly concerning due to the increasing the HIV epidemic, which have increased the risk for reactivation to active tuberculosis (TB) infection. LTBI is diagnosed by tuberculin skin test (TST) and interferon-gamma release assays (IGRAs).

**Objectives:**

The aim of the present study was to conduct a meta-analysis of published papers on the agreement (kappa) between TST and QuantiFERON-TB Gold In-Tube (QFT-GIT) tests for diagnosis of LTBI in HIV patient.

**Methods:**

Electronic databases including PubMed/Medline, Elsevier/Scopus and Embase/Ovid were reviewed up Jan. 2016. We performed a random effect model meta-analysis for estimation of pooled Kappa between the two methods of diagnosis. Meta regression was used for assessing potential heterogeneity and Egger’s test was used for assessing small study effect and publication bias.

**Results:**

The initial search strategy produced 6744 records. Of them, 23 cross-sectional studies met the inclusion criteria and 20 studies entered in meta-analysis. The pooled kappa was and prevalence-adjusted and bias-adjusted kappa (PABAK) were 0.37 (95% CI: 0.28, 0.46) and 0.59 (0.49, 0.69). The discordance of TST-/QFT-GIT+ was more than TST+/QFT-GIT-. Kappa estimate between two tests was linearly associated with age and prevalence index and inversely associated with bias index.

**Conclusion:**

Fair agreement between TST and QFT-GIT makes it difficult to know whether TST is as useful as the QFT-GIT in HIV-infected patients. The higher discordance of TST-/QFT-GIT+ in compared to TST+/QFT-GIT- can induce the higher sensitivity of QFT-GIT for diagnosis LTBI in HIV patients. Disagreement between two tests can be influenced by error in measurements and prevalence of HIV.

## Introduction

Co-infection of Human immunodeficiency virus (HIV) and tuberculosis (TB) is a major public health concern. Recent estimates indicated about 9 million new cases of TB annually worldwide, of which 1.1 million are people living with HIV. Additionally, 1.5 million persons die from TB each year, 360,000 of whom are HIV-positive [1]. It has been shown that the risk of progression from Latent Tuberculosis Infection (LTBI) to active TB is 12 to 20 times greater for people living with HIV than for those without an HIV infection [2]. It has also been identified that most of the deaths resulting from TB in HIV/AIDS patients are preventable if there is an accurate diagnosis and treatment [3]. Tuberculin Skin Test (TST) and Interferon-Gamma Release Assays (IGRAs) are the main tests currently available for the diagnosis of LTBI.

The use of TST which called Mantoux tuberculin test or purified protein derivatives (PPDs), as a standard method of determining *Mycobacterium tuberculosis* infection has some disadvantages: false-positive reactions to infection with *non-tuberculosis mycobacteria* and history of BCG vaccination [4] as well as false-negative reactions in presence of weakened immune system, such as an HIV infection [5]. To deal with the challenges regarding TST, the IGRAs tests are introduced [6]. The QuantiFERON_®_-TB Gold In-Tube test (QFT-GIT) and T-SPOT_®_ TB test (T-Spot) are two IFN-γ release assays (IGRAs) commercially available.

The result of some systematic reviews indicate that in comparison to TST, IGRAs can detect LTBI with a higher specificity, negative (NPV) and positive (PPV) predictive values [7, 8]. The comparative performance of IGRAs and TST in LTBI detection is not clear especially in persons with HIV infection. Ample documents were published on the agreement (kappa) of the QFT-GIT and TST in HIV-positive people; the range of kappa were variable: 0.29 in one study in Georgia [9] and 0.59 in Chile [10]. On the other hand, some factors including gender, age, CD4+ T-cell count can influence the diagnosis of LTBI using TST and/or QFT-GIT [11] and finally on concordance or discordance between the two tests.

The most limitation of the data on the agreement of TST and QFT-GIT in HIV-infected persons is low small sample size [12, 13]. Therefore a pooled study such as meta-analysis using a unique measure with high precision is needed. To the best or our knowledge, there have not been any systematic review and meta-analyses that evaluated the agreement (kappa) between TST and QFT-GIT in LTBI detection among HIV infected people. The aim of this study was to provide reliable evidence and clarify issues regarding this agreement using a systematic review and meta-analysis.

## Materials and Methods

### Search strategy and selection criteria

The systematic review and meta-analysis was conducted according to Preferred Reporting Items for Systematic Review and Meta-analysis (PRISMA) [14]. Electronic databases, including PubMed/Medline, Elsevier/Scopus and Embase/Ovid were searched for published literatures. The following key words were used for search: ‘latent tuberculosis infection’, ‘QuantiFERON’, ‘interferon-gamma release test’, ‘interferon-gamma release assay’, ‘enzyme-linked immunospot assay’, ‘tuberculin test’, ‘PPD-S’, ‘skin test’, ‘mantoux tuberculin skin test’, kappa, kappa-value, kappa-statistic, agreement, observational study.

Reference lists of considered papers, reviews, meta-analyses, letters, and other relevant documents were searched and further communication with the authors of retrieved papers was done for additional information. Primary eligibility criteria for inclusion were: 1) studies that included HIV positive participants, 2) studies that had original data to calculate the kappa coefficient and its standard error. The cut-off value by the manufacturer for QFTGIT is ≥ 0.35 IU/ml and finally blood sampling for QFT-GIT was done before the TST test. Papers were excluded if they: 1) studied HIV people with active TB, 2) studies on agreement between one-step TST with serial QFT-GIT, 3) reviews, cost analyses papers and letters without original data.

### Data extraction and quality assessment

After eliminating duplicate records, two authors (EA and ADA) independently screened the titles for relevance and study selection. Abstracts from selected citations were independently reviewed for further relevance; in cases of disagreement, a third consultant (EM) acted as an arbitrator. The following items were extracted from the included studies and included in a checklist; first author, year of publication, study setting (country), gender, mean age, sample size, the history of BCG vaccination at infancy (yes, no, unknown, non-discrimination), TST cut-off (diameter of induration) as positive, mean/median T-cell CD4 count, and the number of subjects with positive and negative TST/QFT-GIT. Incidence rate of TB per 100, 000 in the study location was extracted from the WHO, Global Tuberculosis Report 2015 [15].

The reporting bias of included studies in the meta-analysis was assessed by a modified checklist from the Strengthening the Reporting of Observational Studies in Epidemiology (STROBE) Statement [16]. The following criteria were assessed; (a) a clear definition of the study population; (b) description of the setting, locations, and relevant dates; (c) an exact definition of the outcome, such as LTBI diagnosis by the TST and/or the QFT-GIT; (d) eligibility criteria for the participants; (e) an explanation of how the study size was determined; (f) figures reflecting the number of outcomes associated with each test; and (g) an explanation of when each test was conducted, such as whether blood sampling for the QFT-GIT took place before the TST. Studies that fulfilled all of the above criteria were classified as having a low risk of bias. Studies that met one criteria were classified as having an intermediate risk of bias, and studies fulfilling more than one criteria were classified as having a high risk of bias.

### Statistical methods

Cohen's kappa statistic, k, is a measure of agreement between categorical variables. Using this statistic, true agreement with accounting the agreement occurring by chance was achieved. Kappa expresses the proportion of agreement beyond that expected by chance. It is defined as the observed agreement not due to chance in relative to the maximum non-chance agreement [17]. Standard error (SE) and a 95% confidence interval (CI) for kappa were calculated using the methods described by J.L. Fleiss et al [18]. To calculate the kappa estimate, positive/negative results for two tests were considered and meaningful cases such as intermediate were discarded. Landis and Koch criteria were applied to interpret the degree of agreement; ≤0: poor, 0.1–0.2: slight, 0.21–0.40: fair, 41–0.60: moderate, 0.61–0.8: substantial and 0.81–1: almost perfect [19]. The inverse variance method was used to estimate the weighted pooled Cohen’s kappa. The I^2^ was used for evaluation between studies heterogeneity.

In the presence of heterogeneity, a random effects model was used to pool the effect estimates. Meta-analysis regression was applied to investigate which factors determine heterogeneity among included individual studies in the meta-analysis. A forest plot was used to show the kappa (95% CI) of individual studies that went into the meta-analysis. The publication bias was evaluated using a funnel plot with the test of Begg et al [20] and the linear regression asymmetry test of Egger et al [21].

Post hoc sensitivity analyses were conducted to estimate the adjusted kappa. It has been shown that the kappa is affected by effect of prevalence and bias. It has been identified that the prevalence of disease and the extent to which the tests disagree on the proportion of positive or negative cases (measurement bias) could influence the kappa coefficient. Prevalence and bias index were calculated as |a-d|/n and |b-c|/n, respectively. It is expressed that when prevalence or bias is low or absent or two latter indexes are large, the kappa will be biased, [22]. PABAK (prevalence-adjusted bias-adjusted kappa) is suggested to simulate when there is no prevalence or no bias effects. An adjustment for prevalence and bias is achieved by substituting ‘the mean of cells a and d’ instead of a and d and ‘the mean of cells b and c’ instead of b and c [23]. The data analyses were performed using Stata software (version 11/SE).

## Results

### The characteristics of included studies

**Fig 1:**
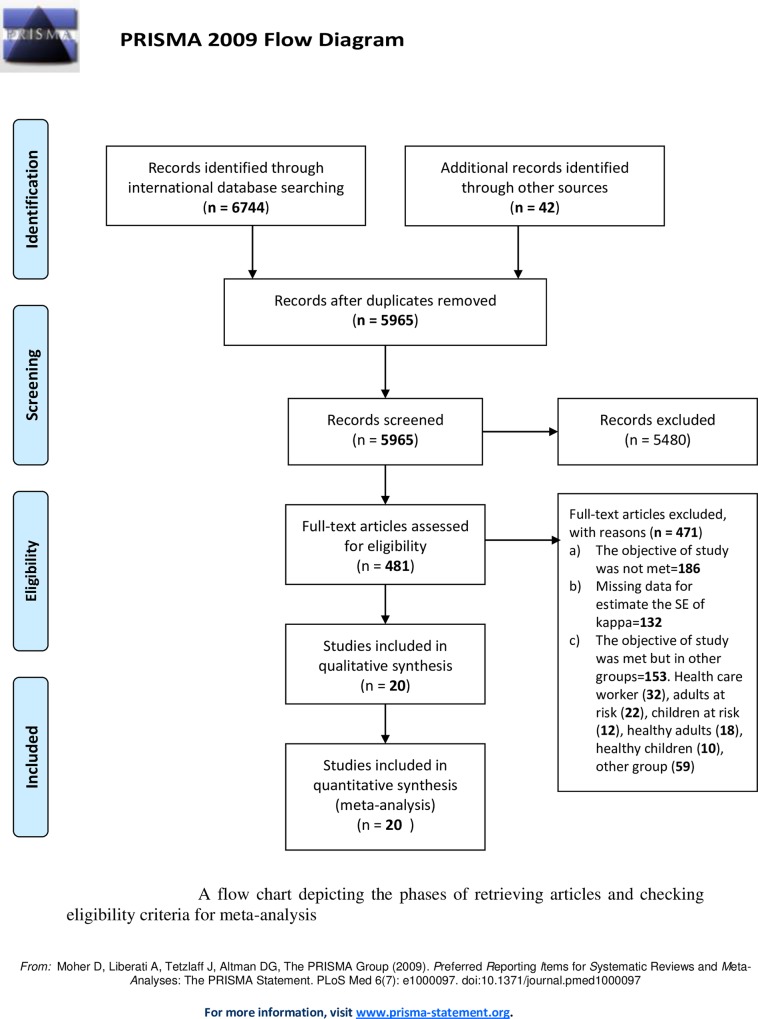
The flow chart of retrieved studies in meta-analysis.

In total, 23 studies fulfilled the inclusion criteria. Twenty records were potentially available for meta-analysis [9–13, 24–38]. Three studies [39–41] fulfilled the eligibility criteria to be included in the meta-analysis but their data were not in usable format to calculate kappa estimate. A PRISMA flow chart, illustrating the details related to the selection process, is presented in **Fig 1**. The characteristics of all included studies are summarized in **Table 1**. The sample sizes of the included studies ranged from 16 to 553 and amounted to 4050 subjects in total. In 13 studies from 20 included studies in meta-analysis, value of TST-/QFT-GIT+ was higher than TST+/QFT-GIT-. The higher difference was observed between value of TST+/QFT-GIT+ and TST-/QFT-GIT-. In One study [36] the values of contingency table was unreported and the S.E. was estimate from width of confidence interval. In the all included studies, a positive TST was defined as ≥ 5mm for HIV-infected patients except in Kabeer BSA et al study [35]. In our study the incidence rate of TB in the all included studies in the Meta analysis was under 22 per 100, 000 population expect for the studies that has been conducted in Tanzania [33], India [35] and Georgia [9] with incidence rate of 327, 167 and 106 per 100, 000 respectively. In line of the WHO high TB burden country lists [15], we divided some included studies in the our meta analysis [9, 33, 35] as studies in the high burden countries and other studies as studies in the low burden countries [10–13, 24–27, 29–31, 34, 37, 38].

**Table 1:** Characteristics of the included studies into meta-analysis.

First author	Publication year	Country	Incidence rate of TB ^a^	Sample size ^b^	Median Age	Men (%)	History of BCG vaccination	Median T-Cell CD4	TST(+), QFT (+)	TST (+), QFT(-)	TST (-), QFT(+)	TST (-), QFT (-)
Aichelburg MC	2014	Austria	7.8	235	40	73	Unknown	376 ^d^	24	3	13	195
Balcells ME	2008	Chile	16	109	38.8	85.3	Mix	393 ^c^^,^ ^e^	9	2	8	90
Cheallaigh C	2013	Ireland	7.4	89	36	59	Mix	338 ^e^	4	5	16	64
Chkhartishvili N	2013	Georgia	106	233	38	66	Mix	255 ^e^	25	16	44	148
Converse PJ	1997	USA	3.1	34		80	Unknown		8	1	9	16
Davarpanah MA	2009	Iran	22	173	38	96.6	Unknown	360 ^d^	40	69	14	50
Garcia EG	2013	Mexico	21	24	34^a^	96	Unknown	364 ^e^	3	0	21	0
Jones S	2007	USA	3.1	191	47.3^a^	51.7	Mix	452 ^c^^,^ ^d^	5	8	6	172
Kabeer BSA	2011	India	167	180	34	62	Unknown		27	6	41	106
Kimura M	1999	USA	3.1	167			Unknown	287 ^e^	9	7	23	128
Luetkemeyer AF	2007	USA	3.1	196	46	78	Unknown	363 ^d^	8	10	11	167
Pullar ND	2014	Norway	8.1	209	40	66	Mix	427 ^e^	34	18	18	139
Ramos JM	2012	Spain	12	345	44	76.9	Mix	470 ^e^	21	25	8	291
Santin M	2011	Spain	12	133	34	77.8	Mix	300 ^d^	7	2	6	118
Sauzullo I	2010	Italy	6	157	39	58	Mix	219 ^e^	25	40	10	82
Seshadri C	2008	Tanzania	327	16	36	34	Mix	382 ^d^	4	3	2	7
Stephan C	2008	Germany	6.2	255	44	81	Mix	408 ^e^	18	14	32	191
Talati N	2009	USA	3.1	273	42^a^	65	Mix	335 ^e^	2	7	5	259
Talati N	2011	Zambia	4.6	298	32	40	Mix					
Weinfurter P	2011	USA	3.1	553		67.8	Mix		4	14	22	513

^a^ incidence rate of TB according to WHO, Global Tuberculosis Report 2015

^b^ the number of subjects that have complete data for two test without intermediate cases

^c^ mean measure

^d^ cell/mm^3^

^e^ cell/μL

### The quality assessment and publication bias

**Fig 2:**
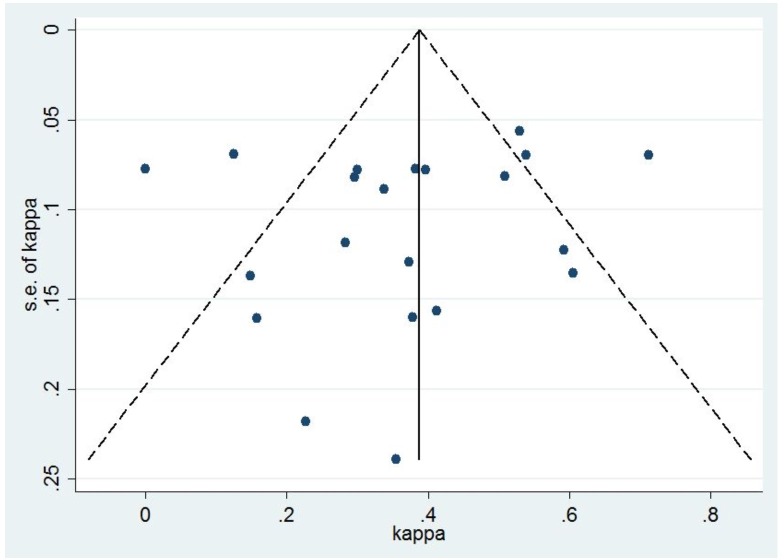
Funnel plot, using data from the 20 included studies in meta-analysis, with kappa displayed on the horizontal axis and S.E. (kappa) on the vertical axis; symmetrical plot shows the absence of publication bias.

Quality assessment of the studies showed four studies of low quality [26, 27, 33, 35], seven intermediate-quality studies [11, 25, 28, 29, 31, 32, 37] and nine high-quality studies [9, 10, 12, 13, 24, 30, 34, 36, 38]. After summation of test results of the included study, it found that discordance of TST-/QFT-GIT+ was more than TST+/QFT-GIT-. (**Table 1**). No evidence of publication bias was found (Egger’s test: p = 0.48) (**Fig 2**).

### Statistical analysis results

**Fig 3:**
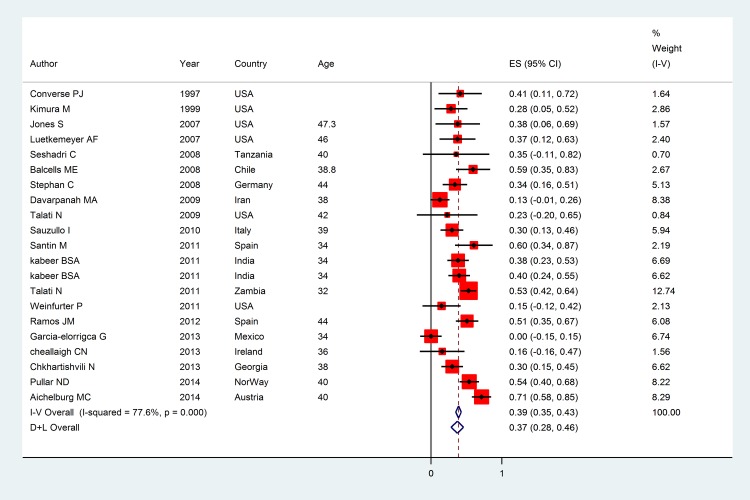
The overall Kappa coefficient: Squares represent kappa from individual studies with 95% confidence intervals; size of square is proportional to the weight assigned to included studies in the meta-analysis. The diamond represents the overall results and 95% confidence interval of the random effect of the meta-analysis.

**Fig 4:**
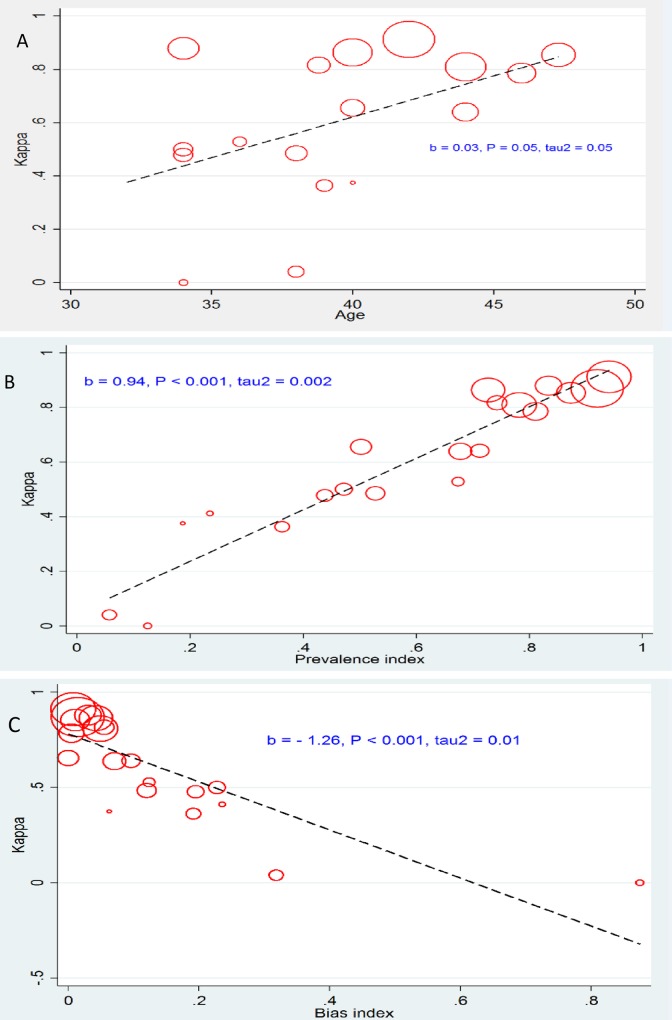
Meta regression plot for effect of explanatory variables on variation of kappa estimate. (A) Age. (B) Prevalence index. (B) Bias index.

**Fig 5:**
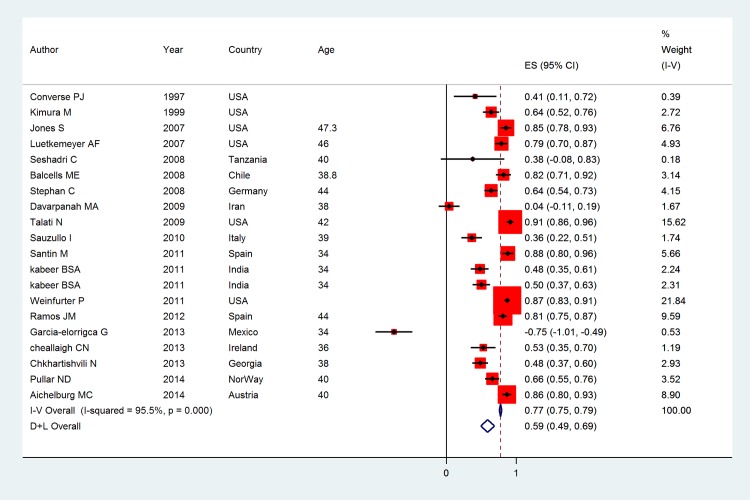
The overall prevalence adjusted bias adjusted kappa coefficient: Squares represent kappa from individual studies with 95% confidence intervals; size of square is proportional to the weight assigned to included studies in the meta-analysis. The diamond represents the overall results and 95% confidence interval of the random effect of the meta-analysis.

The pooled kappa coefficient between TST and QFT-GIT was 0.37 (95% CI: 0.28, 0.46) with the significant heterogeneity was found among studies (I^2^ = 77.6%, p<0.001) (**Fig 3**). Stratified analysis by continents showed that kappa estimate (95%) equal to 0.24 (0.10, 0.36) for North America, 0.44 (0.32, 0.57) for Europe, and 0.52 (0.41, 0.63) and 0.30 (0.12, 0.48) for Africa and Asia. Among studies where some of subjects had a history of BCG vaccination, the kappa estimate was 0.41 (0.33, 0.49) while it was 0.37 (0.28, 0.46) for studies where history of BCG vaccination was unknown. The kappa estimate for high and low burden of TB was 0.36 (0.27, 0.45) and 0.37 (0.26, 0.48) respectively. Based on sub group of quality of studies the result showed that kappa (95%) for low, medium and high quality were 0.34 (0.26, 0.41), 0.80 (0.77, 0.83) and 0.79 (0.77, 0.82) respectively. Meta regression plot showed that age and prevalence index linearly related with kappa and bias index was inversely related **(Fig 4A–4C**). The results suggested that the kappa varied significantly by age, prevalence index and bias index. **Fig 5** illustrated the PABAK estimates of individual studies that the pooled PABAK was 0.59 (0.49, 0.69).

## Discussion

This meta-analysis of the 20 included studies showed that the kappa coefficient between TST and QFT-GIT was fair (0.37). Disagreement (kappa estimate) between TST/QFT-GIT could be attributed to age, country, the prevalence of HIV infection and the bias in measurements.

The major limitation of most of the included studies was low sample size. It has been shown that an unreliable kappa estimate results and the pooled analysis such as meta-analysis was needed to increase the precision using a large sample size. The results of this study showed that the kappa estimate varied across the studies (0 to 0.71). It could be due to test characteristics, study settings and/or subject characteristics. It has been identified that in clinic-based studies the number of QFT-GIT and/or TST-positive subjects was limited [12]. In addition, true prevalence can affect the kappa estimate [42].

In this review, the number of positive QFT-GIT and negative TST, compared to negative QFT-GIT positive TST, was relatively high and this result is in line a meta-analysis that showed QFT-GIT is a more sensitive test in detecting LTBI [7]. One study showed that some factors explain the non-concordance between the two tests, such as co-infections and male gender, and lead to positive TST and positive QFT-GIT, respectively [9]. The high amount of concordance of a negative TST with a negative QFT-G in this study may be ascribed to the prevalence of HIV infection; here most included studies came from areas with low prevalence of infection.

Cattamanchi et al found that an intermediate result for QFT-GIT was <1–11% [43]. This suggested that intermediate results are not associated with degree of immunodeficiency [38, 44], so the intermediate cases of QFT-GIT and TST are meaningfulness with regard to HIV infection. As the number of intermediate cases in studies included in our meta-analysis was trivial, the calculation of the weighted kappa because of loss precision was unwarranted. In this study, as the reversion rate from positive to negative in QFT-GIT serial in HIV-infected patients was identified [45], one step test was considered for two tests to get a valid estimate of agreement.

In this meta-analysis, CD4 counts in all subjects were high. It is identified that when the CD4 count is low, anergic TST responses and low-mitogen QFT-GIT responses occur [10]; the CD4 count may be an effect modifier and the kappa estimate is not uniform within its range. Some studies in countries with a high TB prevalence showed that in individuals with a low CD4+ T-cell count, increased rate of negative QFT occurred [40]. However, one study in Georgia showed that among patients with CD4 counts ≤ 100 μl the IGRAs was more sensitive than TST [9].

It is obvious that imbalance in actual cell or large differences between concordances/no concordance in the included studies was high. It leads to heterogeneity in marginal distribution, so the kappa estimate was far from real value [46]. Sensitivity analysis showed the kappa between the two tests in some area such as North America and Europe could be substantial. It has been criticized using PABAK to simulate the situation of no prevalence and no bias because PABAK can generate a value for kappa that is far from the situation in which the original tests are made [47].

### Limitations of the study

Our study has some limitations that should be considered. The standard errors and confidence interval are generally not reported for kappa in all published studies; this deficit, along with unretrieved the gray literature, could affect real agreement. Uniform data for BCG vaccination and T-cell CD4 count in the included studies were not accessible and their effect on variation between studies were not clear.

## Conclusion

In this study, the pooled kappa estimate was 0.37 (0.28, 0.46). The fair agreement between the two tests makes it unclear which test is optimal to detect LTBI. Age, the prevalence of HIV infection or bias in measurements may be related with agreement between two tests. Further studies are needed to assess the agreement of the two tests in detecting active TB. A network meta-analysis to get valid agreement among TST, QFT-GIT and T-SPOT is recommended.

## Supporting Information

S1 File PRISMA Checklist. (DOCX)

## References

[1] World Health Organization, Geneva: Global tuberculosis control: WHO report 2014: World Health Organization; 2014.

[2] World Health Organization, Geneva: Global tuberculosis control: WHO report 2012. 2012.

[3] Taylor Z, Nolan CM, Blumberg HM. Controlling tuberculosis in the United States. Recommendations from the American Thoracic Society, CDC, and the Infectious Diseases Society of America. MMWR Recommendations and reports: Morbidity and mortality weekly report Recommendations and reports/Centers for Disease Control. 2005;54(RR-12):1–81. 16267499

[4] Farhat M, Greenaway C, Pai M, Menzies D. False-positive tuberculin skin tests: what is the absolute effect of BCG and non-tuberculous mycobacteria?[Review Article]. The International Journal of Tuberculosis and Lung Disease. 2006;10(11):1192–204. 17131776

[5] Lee Y-M, Park K-H, Kim S-M, Park S, Lee S-O, Choi S-H, et al Risk factors for false-negative results of T-SPOT. TB and tuberculin skin test in extrapulmonary tuberculosis. Infection. 2013;41(6):1089–95. 10.1007/s15010-013-0478-z 23943073

[6] Pai M, Riley LW, Colford JM Jr. Interferon-γ assays in the immunodiagnosis of tuberculosis: a systematic review. The Lancet infectious diseases. 2004;4(12):761–76. 15567126 10.1016/S1473-3099(04)01206-X

[7] Diel R, Goletti D, Ferrara G, Bothamley G, Cirillo D, Kampmann B, et al Interferon-γ release assays for the diagnosis of latent Mycobacterium tuberculosis infection: a systematic review and meta-analysis. European Respiratory Journal. 2011;37(1):88–99. 10.1183/09031936.00115110 21030451

[8] Menzies D, Pai M, Comstock G. Meta-analysis: new tests for the diagnosis of latent tuberculosis infection: areas of uncertainty and recommendations for research. Annals of internal medicine. 2007;146(5):340–54. 17339619 10.7326/0003-4819-146-5-200703060-00006

[9] Chkhartishvili N, Kempker RR, Dvali N, Abashidze L, Sharavdze L, Gabunia P, et al Poor agreement between interferon-gamma release assays and the tuberculin skin test among HIV-infected individuals in the country of Georgia. BMC infectious diseases. 2013;13(1):513. 24176032 10.1186/1471-2334-13-513 PMC3817813

[10] Balcells ME, Pérez CM, Chanqueo L, Lasso M, Villanueva M, Espinoza M, et al A comparative study of two different methods for the detection of latent tuberculosis in HIV-positive individuals in Chile. International journal of infectious diseases. 2008;12(6):645–52. 10.1016/j.ijid.2008.03.005 18534887

[11] Ramos JM, Robledano C, Masiá M, Belda S, Padilla S, Rodríguez JC, et al Contribution of Interferon gamma release assays testing to the diagnosis of latent tuberculosis infection in HIV-infected patients: A comparison of QuantiFERON-TB gold in tube, T-SPOT. TB and tuberculin skin test. BMC infectious diseases. 2012;12(1):169. 22849726 10.1186/1471-2334-12-169 PMC3482589

[12] Aichelburg M, Mandorfer M, Tittes J, Breitenecker F, Reiberger T, Rieger A, et al The association of smoking with IGRA and TST results in HIV-1-infected subjects. The International Journal of Tuberculosis and Lung Disease. 2014;18(6):709–16. 10.5588/ijtld.13.0813 24903943

[13] Santin M, Casas S, Saumoy M, Andreu A, Moure R, Alcaide F, et al Detection of latent tuberculosis by the tuberculin skin test and a whole-blood interferon-γ release assay, and the development of active tuberculosis in HIV-seropositive persons. Diagnostic microbiology and infectious disease. 2011;69(1):59–65. 10.1016/j.diagmicrobio.2010.09.005 21146715

[14] Moher D, Liberati A, Tetzlaff J, Altman DG. Preferred reporting items for systematic reviews and meta-analyses: the PRISMA statement. Annals of internal medicine. 2009;151(4):264–9. 19622511 10.7326/0003-4819-151-4-200908180-00135

[15] WHO. Tuberculosis Country Profiles. [07 August 2016, date last accessed]. Available from: http://www.who.int/tb/country/data/profiles/en/.

[16] von Elm E, Altman DG, Egger M, Pocock SJ, Gotzsche PC, Vandenbroucke JP. Strengthening the Reporting of Observational Studies in Epidemiology (STROBE) statement: guidelines for reporting observational studies. BMJ. 2007;335(7624):806–8. 17947786 10.1136/bmj.39335.541782.AD PMC2034723

[17] Cohen J. A coefficient of agreement for nominal scales. educ psychol meas. 1960;20:37–46.

[18] Fleiss JL, Levin B, Paik MC. Statistical methods for rates and proportions: John Wiley & Sons; 2013.

[19] Landis JR, Koch GG. The measurement of observer agreement for categorical data. biometrics. 1977:159–74. 843571

[20] Begg CB, Mazumdar M. Operating characteristics of a rank correlation test for publication bias. Biometrics. 1994:1088–101. 7786990

[21] Egger M, Smith GD, Schneider M, Minder C. Bias in meta-analysis detected by a simple, graphical test. Bmj. 1997;315(7109):629–34. 9310563 10.1136/bmj.315.7109.629 PMC2127453

[22] Sim J, Wright CC. The kappa statistic in reliability studies: use, interpretation, and sample size requirements. Physical therapy. 2005;85(3):257–68. 15733050

[23] Byrt T, Bishop J, Carlin JB. Bias, prevalence and kappa. Journal of clinical epidemiology. 1993;46(5):423–9. 8501467 10.1016/0895-4356(93)90018-v

[24] Cheallaigh CN, Fitzgerald I, Grace J, Singh GJ, El-Eraki N, Gibbons N, et al Interferon gamma release assays for the diagnosis of latent TB infection in HIV-infected individuals in a low TB burden country. PloS one. 2013;8(1):e53330 10.1371/journal.pone.0053330 23382842 PMC3559731

[25] Converse PJ, Jones SL, Astemborski J, Vlahov D, Graham NM. Comparison of a tuberculin interferon-γ assay with the tuberculin skin test in high-risk adults: effect of human immunodeficiency virus infection. Journal of Infectious Diseases. 1997;176(1):144–50. 9207360 10.1086/514016

[26] Davarpanah M, Rasti M, Mehrabani D, Allahyari S, Neirami R, Saberi-Firoozi M. Association between PPD and QuantiFERON Gold TB test in TB infection and disease among HIV-infected individuals in southern Iran. Iranian Red Crescent Medical Journal. 2009;11(1):71–5.

[27] Garcia-Elorriaga G, Martinez-Velazquez M, Gaona-Flores V, del Rey-Pineda G, Gonzalez-Bonilla C. Interferon gamma in patients with HIV/AIDS and suspicion or latent tuberculosis infection. Asian Pacific journal of tropical medicine. 2013;6(2):135–8. 10.1016/S1995-7645(13)60009-7 23339916

[28] Jones S, De Gijsel D, Wallach F, Gurtman A, Shi Q, Sacks H. Utility of QuantiFERON-TB Gold in-tube testing for latent TB infection in HIV-infected individuals. The international journal of tuberculosis and lung disease: the official journal of the International Union against Tuberculosis and Lung Disease. 2007;11(11):1190–5. 17958980

[29] Kimura M, Converse PJ, Astemborski J, Rothel JS, Vlahov D, Comstock GW, et al Comparison between a whole blood interferon-gamma release assay and tuberculin skin testing for the detection of tuberculosis infection among patients at risk for tuberculosis exposure. The Journal of infectious diseases. 1999;179(5):1297–300. . 10191241 10.1086/314707

[30] Luetkemeyer AF, Charlebois ED, Flores LL, Bangsberg DR, Deeks SG, Martin JN, et al Comparison of an interferon-γ release assay with tuberculin skin testing in HIV-infected individuals. American journal of respiratory and critical care medicine. 2007;175(7):737–42. 17218620 10.1164/rccm.200608-1088OC PMC1899289

[31] Pullar N, Steinum H, Tonby K, Heggelund L, Leiva R, Ofstad R, et al Low prevalence of positive interferon-gamma tests in HIV-positive long-term immigrants in Norway. The International Journal of Tuberculosis and Lung Disease. 2014;18(2):180–7. 10.5588/ijtld.13.0276 24429310

[32] Sauzullo I, Mengoni F, Scrivo R, Valesini G, Potenza C, Skroza N, et al Evaluation of QuantiFERON®-TB Gold In-Tube in human immunodeficiency virus infection and in patient candidates for anti-tumour necrosis factor-alpha treatment. The International Journal of Tuberculosis and Lung Disease. 2010;14(7):834–40. 20550765

[33] Seshadri C, Uiso LO, Ostermann J, Diefenthal H, Shao HJ, Chu HY, et al Low sensitivity of T-cell based detection of tuberculosis among HIV co-infected Tanzanian inpatients. East African medical journal. 2008;85(9):442 19537417 10.4314/eamj.v85i9.117085 PMC3168735

[34] Stephan C, Wolf T, Goetsch U, Bellinger O, Nisius G, Oremek G, et al Comparing QuantiFERON-tuberculosis gold, T-SPOT tuberculosis and tuberculin skin test in HIV-infected individuals from a low prevalence tuberculosis country. Aids. 2008;22(18):2471–9. 10.1097/QAD.0b013e3283188415 19005270

[35] Syed Ahamed Kabeer B, Sikhamani R, Raja A. Comparison of interferon gamma–inducible protein-10 and interferon gamma–based QuantiFERON TB Gold assays with tuberculin skin test in HIV-infected subjects. Diagnostic microbiology and infectious disease. 2011;71(3):236–43. 10.1016/j.diagmicrobio.2011.07.012 21996360 PMC3193601

[36] Talati NJ, Gonzalez-Diaz E, Mutemba C, Wendt J, Kilembe W, Mwananyanda L, et al Diagnosis of latent tuberculosis infection among HIV discordant partners using interferon gamma release assays. BMC infectious diseases. 2011;11(1):264. 21962029 10.1186/1471-2334-11-264 PMC3198954

[37] Talati NJ, Seybold U, Humphrey B, Aina A, Tapia J, Weinfurter P, et al Poor concordance between interferon-γ release assays and tuberculin skin tests in diagnosis of latent tuberculosis infection among HIV-infected individuals. BMC infectious diseases. 2009;9(1):15. 19208218 10.1186/1471-2334-9-15 PMC2649136

[38] Weinfurter P, Blumberg H, Goldbaum G, Royce R, Pang J, Tapia J, et al Predictors of discordant tuberculin skin test and QuantiFERON®-TB Gold In-Tube results in various high-risk groups. The International Journal of Tuberculosis and Lung Disease. 2011;15(8):1056–61. 10.5588/ijtld.10.0650 21740668

[39] Mandalakas A, Hesseling A, Chegou N, Kirchner H, Zhu X, Marais B, et al High level of discordant IGRA results in HIV-infected adults and children. The International Journal of Tuberculosis and Lung Disease. 2008;12(4):417–23. 18371268

[40] Rangaka MX, Wilkinson KA, Seldon R, Van Cutsem G, Meintjes GA, Morroni C, et al Effect of HIV-1 infection on T-Cell–based and skin test detection of tuberculosis infection. American journal of respiratory and critical care medicine. 2007;175(5):514–20. 17158278 10.1164/rccm.200610-1439OC

[41] Richeldi L, Losi M, D'Amico R, Luppi M, Ferrari A, Mussini C, et al Performance of tests for latent tuberculosis in different groups of immunocompromised patients. CHEST Journal. 2009;136(1):198–204. 10.1378/chest.08-2575 19318676

[42] Banerjee M, Fielding J. Interpreting kappa values for two‐observer nursing diagnosis data. Research in nursing & health. 1997;20(5):465–70. 9334800 10.1002/(sici)1098-240x(199710)20:5<465::aid-nur10>3.0.co;2-8

[43] Cattamanchi A, Smith R, Steingart KR, Metcalfe JZ, Date A, Coleman C, et al Interferon-gamma release assays for the diagnosis of latent tuberculosis infection in HIV-infected individuals–a systematic review and meta-analysis. Journal of acquired immune deficiency syndromes (1999). 2011;56(3):230. 21239993 10.1097/QAI.0b013e31820b07ab PMC3383328

[44] Oni T, Gideon HP, Bangani N, Tsekela R, Seldon R, Wood K, et al Risk factors associated with indeterminate gamma interferon responses in the assessment of latent tuberculosis infection in a high-incidence environment. Clinical and Vaccine Immunology. 2012;19(8):1243–7. 10.1128/CVI.00166-12 22718129 PMC3416070

[45] Gray J, Reves R, Johnson S, Belknap R. Identification of false-positive QuantiFERON-TB Gold In-Tube assays by repeat testing in HIV-infected patients at low risk for tuberculosis. Clinical Infectious Diseases. 2012;54(3):e20–e3. 10.1093/cid/cir792 22057704

[46] Blackman NJM, Koval JJ. Interval estimation for Cohen's kappa as a measure of agreement. Statistics in medicine. 2000;19(5):723–41. 10700742 10.1002/(sici)1097-0258(20000315)19:5<723::aid-sim379>3.0.co;2-a

[47] Hoehler FK. Bias and prevalence effects on kappa viewed in terms of sensitivity and specificity. Journal of clinical epidemiology. 2000;53(5):499–503. 10812322 10.1016/s0895-4356(99)00174-2

