# Correction: The Clinical Usefulness of Tuberculin Skin Test versus Interferon-Gamma Release Assays for Diagnosis of Latent Tuberculosis in HIV Patients: A Meta-Analysis

**DOI:** 10.1371/journal.pone.0165143

**Published:** 2016-10-18

**Authors:** The PLOS ONE Staff

There are errors in the author affiliations. The affiliations should appear as shown here:

Erfan Ayubi^1,2,3^, Amin Doosti-Irani^2,3^, Ali Sanjari Moghaddam^4^, Mohadeseh Sani^5^, Milad Nazarzadeh^6^, Ehsan Mostafavi^2,7^*

**1** Department of Epidemiology, School of Public Health, Shahid Beheshti University of Medical Sciences, Tehran, Iran, **2** Department of Epidemiology, Pasteur Institute of Iran, Tehran, Iran, **3** Department of Epidemiology and Biostatistics, School of Public Health, Tehran University of Medical Sciences, Tehran, Iran, **4** School of Medicine, Shahid Beheshti University of Medical Sciences, Tehran, Iran, **5** School of Medicine, Zabol University of Medical Sciences, Zabol, Iran, **6** The collaboration center of meta-analysis research (ccMETA), Iranian Research Center on Healthy Aging, Sabzevar University of Medical Sciences, Sabzevar, Iran, **7** Research Centre for Emerging and Reemerging infectious diseases, Pasteur Institute of Iran, Akanlu, Kabudar Ahang, Hamadan, Iran

There are errors in the Corresponding Author’s email address. The publisher apologizes for the error.

The email address should appear as shown here:

* mostafavi@pasteur.ac.ir, mostafaviehsan@gmail.com
