# Supplementary material for: The Clinical Usefulness of Tuberculin Skin Test versus Interferon-Gamma Release Assays for Diagnosis of Latent Tuberculosis in HIV Patients: A Meta-Analysis
Source: PLoS One. 2016 Sep 13;11(9):e0161983. doi: 10.1371/journal.pone.0161983 (PMC5021339; doi:10.1371/journal.pone.0161983)
Supplement: S1 File — (DOCX) [file pone.0161983.s001.docx]

| **Section/topic** | **#** | **Checklist item** | **Reported on page #** |
| --- | --- | --- | --- |
| **TITLE** | | |  |
| Title | 1 | The clinical usefulness of Tuberculin skin test versus interferon-gamma release assays for diagnosis of latent Tuberculosis in HIV patients: A meta-analysis | 1 |
| **ABSTRACT** | | |  |
| Structured summary | 2 | A complete structured summary is provided on page 2 and 3 | 2-3 |
| **INTRODUCTION** | | |  |
| Rationale | 3 | The most limitation of the data on the agreement of TST and QFT-GIT in HIV-infected persons is low small sample size. Therefore, a pooled study such as meta-analysis using a unique measure with high precision is needed. To the best of our knowledge, there have not been any systematic review and meta-analyses that evaluated the agreement (kappa) between TST and QFT-GIT in LTBI detection among HIV infected people. The frequent application of kappa allows conducting the meta-analyses to examine generalizability of reliability across multiple studies. | 4-5 |
| Objectives | 4 | The aim of this study was to provide reliable evidence and clarify issues regarding this agreement using a systematic review and meta-analysis | 4-5 |
| **METHODS** | | |  |
| Protocol and registration | 5 | The systematic review and meta-analysis was conducted according to Preferred Reporting Items for Systematic Review and Meta-analysis (PRISMA) | 6 |
| Eligibility criteria | 6 | Reference lists of considered papers, reviews, meta-analyses, letters, and other relevant documents were searched and further communication with the authors of retrieved papers was done for additional information. Primary eligibility criteria for inclusion were: 1) studies that included HIV positive participants, 2) studies that had original data to calculate the kappa coefficient and its standard error. The cut-off value by the manufacturer for QFTGIT is ≥ 0.35 IU/ml and finally blood sampling for QFT-GIT was done before the TST test. Papers were excluded if they: 1) studied HIV people with active TB, 2) studies on agreement between one-step TST with serial QFT-GIT, 3) reviews, cost analyses papers and letters without original data | 6 |
| Information sources | 7 | Electronic databases, including PubMed/Medline, Elsevier/Scopus and Embase/Ovid were searched for published literature. | 6 |
| Search | 8 | The following key words were used for search: ‘latent tuberculosis infection’, ‘QuantiFERON’, ‘interferon-gamma release test’, ‘interferon-gamma release assay’, ‘enzyme-linked immunospot assay’, ‘tuberculin test’, ‘PPD-S’, ‘skin test’, ‘mantoux tuberculin skin test’, kappa, kappa-value, kappa-statistic, agreement, observational study. Reference lists of considered papers, reviews, meta-analyses, letters, and other relevant documents were searched and further communication with the authors of retrieved papers was done for additional information. | 6 |
| Study selection | 9 | Fig 1. |  |
| Data collection process | 10 | After eliminating duplicate records, two authors (EA and ADA) independently screened the titles for relevance and study selection. Abstracts from selected citations were independently reviewed for further relevance; in cases of disagreement, a third consultant (EM) acted as an arbitrator | 6 |
| Data items | 11 | The following items were extracted from the included studies and included in a checklist; first author, year of publication, study setting (country), gender, mean age, sample size, the history of BCG vaccination at infancy (yes, no, unknown, non-discrimination), TST cut-off (diameter of induration) as positive, mean/median T-cell CD4 count, and the number of subjects with positive and negative TST/QFT-GIT. | 6-7 |
| Risk of bias in individual studies | 12 | The reporting bias of included studies in the meta-analysis was assessed by a modified checklist from the Strengthening the Reporting of Observational Studies in Epidemiology (STROBE) Statement. The following criteria were assessed; (a) a clear definition of the study population; (b) description of the setting, locations, and relevant dates; (c) an exact definition of the outcome, such as LTBI diagnosis by the TST and/or the QFT-GIT; (d) eligibility criteria for the participants; (e) an explanation of how the study size was determined; (f) figures reflecting the number of outcomes associated with each test; and (g) an explanation of when each test was conducted, such as whether blood sampling for the QFT-GIT took place before the TST . Studies that fulfilled all of the above criteria were classified as having a low risk of bias. Studies that met one criteria were classified as having an intermediate risk of bias, and studies fulfilling more than one criteria were classified as having a high risk of bias. | 7 |
| Summary measures | 13 | **Section:** Statistical analysis | 7-8 |
| Synthesis of results | 14 | **Section:** Statistical analysis | 7-8 |

Page 1 of 2

| **Section/topic** | **#** | **Checklist item** | **Reported on page #** |
| --- | --- | --- | --- |
| Risk of bias across studies | 15 | The publication bias was evaluated using a funnel plot with the test of Begg et al and the linear regression asymmetry test of Egger et al. | 8 |
| Additional analyses | 16 | **Section:** Statistical analysis | 8 |
| **RESULTS** | | |  |
| Study selection | 17 | In total, 23 studies fulfilled the inclusion criteria. Twenty records were potentially available for meta-analysis. Three studies. fulfilled the eligibility criteria to be included in the meta-analysis but their data were not in usable format to calculate kappa estimate. A PRISMA flow chart, illustrating the details related to the selection process, is presented in Fig 1 | 9 |
| Study characteristics | 18 | The characteristics of all included studies are summarized in Table 1. The sample sizes of the included studies ranged from 16 to 553 and amounted to 4050 subjects in total. In 13 studies from 20 included studies in meta-analysis, value of TST-/QFT-GIT+ was higher than TST+/QFT-GIT-. The higher difference was observed between value of TST+/QFT-GIT+ and TST-/QFT-GIT-. In One study the values of contingency table was unreported and the S.E. was estimate from width of confidence interval | 9 |
| Risk of bias within studies | 19 | Quality assessment of the studies showed four studies of low quality, seven intermediate-quality studies and nine high-quality studies. After summation of test results of the included study, it found that discordance of TST-/QFT-GIT+ was more than TST+/QFT-GIT- (Table 1). | 9-10 |
| Results of individual studies | 20 | Results of individual studies has depicted in table1. |  |
| Synthesis of results | 21 | The pooled kappa coefficient between TST and QFT-GIT was 0.37 (95% CI: 0.28, 0.46) with the significant heterogeneity was found among studies (I2 = 77.6%, p<0.001) (Fig 2). Stratified analysis by continents showed that kappa estimate (95%) equal to 0.24 (0.10, 0.36) for North America, 0.44 (0.32, 0.57) for Europe, and 0.52 (0.41, 0.63) and 0.30 (0.12, 0.48) for Africa and Asia. Among studies where some of subjects had a history of BCG vaccination, the kappa estimate was 0.41 (0.33, 0.49) while it was 0.37 (0.28, 0.46) for studies where BCG vaccination was unknown. Based on sub group of quality of studies the result showed that kappa (95%) for low, medium and high quality were 0.34 (0.26, 0.41), 0.80 (0.77, 0.83) and 0.79 (0.77, 0.82) respectively. Meta regression plot showed that age and prevalence index linearly related with kappa and bias index was inversely related (Fig 3a to 3c). The results suggested that the kappa varied significantly by age, prevalence index and bias index. Fig 4 illustrated the PABAK estimates of individual studies that the pooled PABAK was 0.59 (0.49, 0.69). | 10 |
| Risk of bias across studies | 22 | No evidence of publication bias was found (Egger’s test: p=0.48) (Fig 5). | 10 |
| Additional analysis | 23 | Stratified analysis by continents showed that kappa estimate (95%) equal to 0.24 (0.10, 0.36) for North America, 0.44 (0.32, 0.57) for Europe, and 0.52 (0.41, 0.63) and 0.30 (0.12, 0.48) for Africa and Asia. Among studies where some of subjects had a history of BCG vaccination, the kappa estimate was 0.41 (0.33, 0.49) while it was 0.37 (0.28, 0.46) for studies where BCG vaccination was unknown. Based on sub group of quality of studies the result showed that kappa (95%) for low, medium and high quality were 0.34 (0.26, 0.41), 0.80 (0.77, 0.83) and 0.79 (0.77, 0.82) respectively | 10 |
| **DISCUSSION** | | |  |
| Summary of evidence | 24 | This meta-analysis of the 20 included studies showed that the kappa coefficient between TST and QFT-GIT was fair (0.37). Disagreement (kappa estimate) between TST/QFT-GIT could be attributed to age, country, the prevalence of HIV infection and the bias in measurements. | 11 |
| Limitations | 25 | Our study has some limitations that should be considered. The standard errors and confidence interval are generally not reported for kappa in all published studies; this deficit, along with unretrieved the gray literature, could affect real agreement. Uniform data for BCG vaccination and T-cell CD4 count in the included studies were not accessible and their effect on variation between studies were not clear. | 12 |
| Conclusions | 26 | In this study, the pooled kappa estimate was 0.37 (0.28, 0.46). The fair agreement between the two tests makes it unclear which test is optimal to detect LTBI. Age, the prevalence of HIV infection or bias in measurements may be related with agreement between two tests. Further studies are needed to assess the agreement of the two tests in detecting active TB. A network meta-analysis to get valid agreement among TST, QFT-GIT and T-SPOT is recommended | 13 |
| **FUNDING** | | |  |
| Funding | 27 |  |  |

*From:*  Moher D, Liberati A, Tetzlaff J, Altman DG, The PRISMA Group (2009). Preferred Reporting Items for Systematic Reviews and Meta-Analyses: The PRISMA Statement. PLoS Med 6(7): e1000097. doi:10.1371/journal.pmed1000097
